# SAMD9 Promotes Postoperative Recurrence of Esophageal Squamous Cell Carcinoma by Stimulating MYH9‐Mediated GSK3*β*/*β*‐Catenin Signaling

**DOI:** 10.1002/advs.202203573

**Published:** 2023-02-09

**Authors:** Qing Li, Hao Luo, Fu‐Qiang Dai, Ren‐Tao Wang, Xiao‐Qing Fan, Yuan‐Yuan Luo, Meng‐Sheng Deng, Yulun Wang, Tan Long, Wei Guo, Bo Xu, Cheng‐Xiong Xu, Hua Jin

**Affiliations:** ^1^ Department of Thoracic Surgery Daping Hospital Army Medical University Chongqing 400042 China; ^2^ Cancer Center Daping Hospital Army Medical University Chongqing 400042 China; ^3^ College of Pulmonary and Critical Care Medicine Chinese PLA General Hospital Beijing 100853 China; ^4^ School of Medicine Chongqing University Chongqing 400030 China; ^5^ State Key Laboratory of Trauma Research Institute of Surgery Army Medical University Chongqing 400042 China; ^6^ Department of Biochemistry and Molecular Biology National Clinical Research Center for Cancer Key Laboratory of Cancer Prevention and Therapy Tianjin Tianjin's Clinical Research Center for Cancer Tianjin 300060 China; ^7^ Chongqing Key Laboratory of Intelligent Oncology for Breast Cancer Chongqing University Cancer Hospital and Chongqing University School of Medicine Chongqing 400030 China

**Keywords:** esophageal squamous cell carcinoma recurrence, myosin‐9, sterile alpha motif domain‐containing protein 9, *β*‐catenin signaling

## Abstract

Recurrence is a challenge to survival after the initial treatment of esophageal squamous cell carcinoma (ESCC). But, its mechanism remains elusive and there are currently no biomarkers to predict postoperative recurrence. Here, the possibility of sterile alpha motif domain‐containing protein 9 (SAMD9) as a predictor of postoperative recurrence of ESCC is evaluated and the molecular mechanisms by which SAMD9 promotes ESCC recurrence are elucidated. The authors found that the high level of SAMD9 is correlated with postoperative recurrence and poor prognosis of ESCC. Overexpression of SAMD9 promotes tumor stemness, angiogenesis, and EMT, while downregulation of SAMD9 reduced these phenotypes. Mechanistically, it is found that SAMD9 stimulated ubiquitination‐mediated glycogen synthase kinase‐3 beta (GSK‐3*β*) degradation by interaction with myosin‐9 (MYH9) and TNF receptor‐associated factor 6 (TRAF6), which in turn activated Wnt/*β*‐catenin pathway. Further, the authors demonstrated that silencing SAMD9 inhibited lung metastasis and tumor formation in vivo. Finally, the authors found that silencing MYH9 or *β*‐catenin, or overexpressing GSK‐3*β* inhibited SAMD9‐stimulated ESCC cell stemness, EMT, angiogenesis, metastasis, and tumorigenicity. Together, the findings indicate that the SAMD9/MYH9/GSK3*β*/*β*‐catenin axis promotes ESCC postoperative recurrence and that SAMD9 is a crucial target for ESCC therapy. Additionally, SAMD9 has the potential as a predictor of postoperative recurrence in ESCC.

## Graphical abstract

This work demonstrates the clinical and functional role of SAMD9 in esophageal squamous cell carcinoma (ESCC). SAMD9 promotes MYH9/TRAF6‐regulated ubiquitination and degradation of GSK3*β* by binding to MYH9, leading to activation of the *β*‐catenin signaling pathway, which stimulates EMT, angiogenesis, and stemness of ESCC cells, and ultimately promotes ESCC metastasis and recurrence.

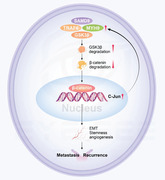

## 1 Introduction

The mortality of patients with esophageal cancer (ES) ranks sixth among cancers in the world,^[^ ^1^ ^]^ and its main histological subtype is esophageal squamous cell carcinoma (ESCC).^[^ ^2^ ^]^ Surgery remains to be the major treatment method for ESCC. Unfortunately, 74% of ESCC patients had metastasis after surgery, and the survival rate was only 39.8%.^[^ ^2^ ^]^ According to a previous report, even in early‐stage ESCC patients with negative lymph nodes’ involvement, the postoperative recurrence is as high as 40% (including local and metastatic recurrence),^[^ ^3^ ^]^ indicating that recurrence is a major challenge in ESCC treatment. However, there are no biomarkers to predict the postoperative recurrence of ESCC, and the recurrence mechanism remains unclear.

Previous studies have shown that sterile alpha motif domain‐containing protein 9 (SAMD9) is associated with the progression of several cancers.^[^ ^4^, ^6^ ^]^ However, studies show that SAMD9 plays opposite roles in different cancers. For example, SAMD9‐overexpression suppresses non‐small cell lung cancer progression,^[^ ^4^ ^]^ while it promotes disease progression in glioblastoma.^[^ ^5^ ^]^ Importantly, a report has shown that SAMD9 is upregulated in primary tumors of ESCC patients with metastasis compared to no metastasis,^[^ ^6^ ^]^ suggesting that SAMD9 may be involved in promoting ESCC metastasis. But, the tumor‐promoting mechanism of SAMD9 in ESCC is unclear, and the correlation between SAMD9 and postoperative recurrence in ESCC has not been reported.

Myosin‐9 (YH9) is a non‐muscle myosin heavy chain IIA involved in the regulation of cell migration.^[^ ^7^ ^]^ Studies have shown that MYH9 is aberrantly upregulated and it enhances cancer stemness in gastric^[^ ^8^ ^]^ and liver cancers.^[^ ^9^ ^]^ Importantly, cancer stemness is a crucial factor for cancer metastatic recurrence.^[^ ^10^ ^]^ Upregulated MYH9 expression and its correlation with metastasis in ESCC was reported previously.^[^ ^11^ ^]^ These findings suggest that MYH9 might promote ESCC metastasis by enhancing cancer stemness, but whether MYH9 promotes ESCC progression through cancer stemness has not been reported. Additionally, the mechanism leading to the overexpression of MYH9 in ESCC is also unclear.

Here, we report that SAMD9 high expression is closely associated with the postoperative recurrence in ESCC and that SAMD9‐overexpression promotes the tumorigenicity and metastasis of ESCC cells by stimulating cancer stemness, angiogenesis, and EMT. In contrast, silencing of SAMD9 inhibited metastasis and tumorigenicity of ESCC cells. In addition, we demonstrated that SAMD9 stimulates MYH9 expression, cancer stemness, angiogenesis, and EMT by activating the *β*‐catenin pathway. Finally, we found that SAMD9 enhances the interaction between TRAF6 and GSK3*β* through binding to MYH9, thereby promoting the ubiquitination of GSK3*β* by TRAF6, further stimulating *β*‐catenin signaling. This study elucidates a novel mechanism for the postoperative recurrence of ESCC and provides a new target for ESCC treatment.

## 2 Results

### 2.1 SAMD9 Expression is Associated with Postoperative Recurrence of ESCC

**Figure 1:**
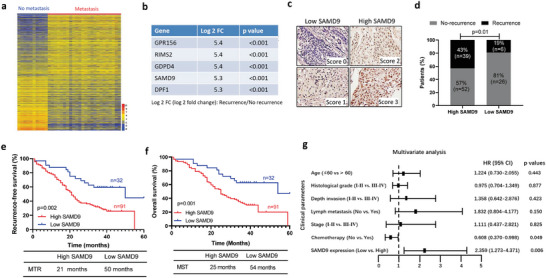
High level of SAMD9 is correlated with postoperative recurrence of ESCC. a) Heatmap showing differentially expressed genes between primary tumors of ESCC patients with postoperative metastatic recurrence and no recurrence. RNA sequencing was performed using primary tumors from ESCC patients with metastatic recurrence within 24 months after operation (*n* = 24) and no recurrence within 60 months after operation (*n* = 10). b) Genes that are increased in primary tumors from ESCC patients who had metastatic recurrence compared to those who had no recurrence. c) SAMD9 expression in primary tumors of ESCC patients was measured by immunohistochemistry (IHC) and the IHC staining was scored. Scores 0 and 1 were defined as low expression, and scores 2 and 3 were defined as high expression. d) The SAMD9 expression level is associated with postoperative recurrence of ESCC. Based on IHC scores, 123 ESCC cases were divided into high‐ and low‐ expression groups of SAMD9, and then, demonstrated the correlation between SAMD9 expression level and recurrence in ESCC patients. *p‐*value was calculated by the chi‐square test. e,f) Kaplan–Meier survival analysis shows the high level of SAMD9 was associated with lower recurrence‐free survival rate and a lower 5‐year overall survival rate of ESCC. *p‐*value was calculated by the log‐rank test. g) Multivariate survival analyses of clinicopathological characteristics indicate that SAMD9 is an independent poor prognostic factor of ESCC (*n* = 123). MTR, median time to recurrence; MST, median survival time. *p‐*values less than 0.05 were considered statistically significant.

To identify transcriptomic signature associated with ESCC recurrence, RNA sequencing (**Figure** **1**a) was performed using primary tumors from patients with metastatic recurrence within 24 months after operation (*n* = 24) and those without recurrence within 60 months after operation (*n* = 10) (Table S1, Supporting Information). As shown in Figure 1b, the top five genes, including GPR156, RIMS2, GDPD4, SAMD9, and DPF1, showed significantly higher expression in primary tumors of ESCC patients with metastatic recurrence compared to that without recurrence. Since SAMD9 was previously shown to be upregulated in primary tumors of ESCC patients with metastasis compared to no metastasis, we focused on validating SAMD9 in ESCC metastatic recurrence. The correlation between the SAMD9 level and postoperative recurrence was confirmed in additional ESCC patients. Based on immunohistochemistry (IHC) scoring of the primary tumor, 123 ESCC patients were assigned to high and low SAMD9 expression groups (Figure 1c). We found that 43% of the cases in the high SAMD9 expression group had postoperative recurrence, accounting for 87% of the total recurrence, while only 19% of cases in the low SAMD9 group had recurrence, accounting for 13% of the total recurrence (Figure 1d). Additionally, patients in the high SAMD9 group had lower recurrence‐free survival (RFS) and overall survival (Figure 1e,f). The median time to recurrence (MTR) and median survival time (MST) of these patients in the SAMD9 high expression group were 21 and 25 months respectively, which were significantly shorter than 50 months of MTR and 54 months of MST in the SAMD9 low expression group (Figure 1e,f). Finally, we found that a high level of SAMD9 is an independent indicator of ESCC overall survival (Figure 1g). These data indicate that SAMD9 level is positively correlated to postoperative recurrence and poor prognosis of ESCC.

### 2.2 SAMD9 Enhances Tumor Stemness and Metastasis Ability of ESCC Cells

**Figure 2:**
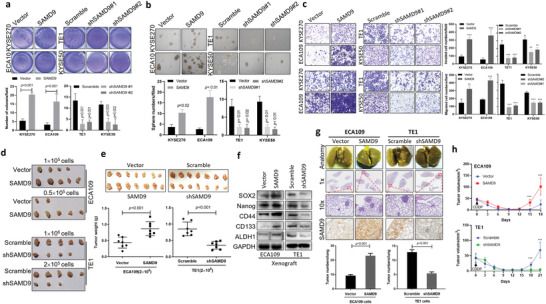
SAMD9 promotes tumorigenicity and metastasis ability of ESCC cells. a) SAMD9 promotes soft agar colony formation, b) sphere formation, c) invasion and migration of ESCC cells. After 24 h of transfection with the indicated constructs, cells were subjected to analysis. Each bar represents the mean of three independent experiments. d) A subcutaneous xenograft model experiments showing that SAMD9 positively regulates ESCC cells tumorigenicity (*n* = 5/group) and e) tumor growth (*n* = 7/group). The indicated amounts of cells in 0.1 mL PBS were injected subcutaneously into the back of the mice. One month after cell injection, mice were sacrificed and tumors were collected. f) Western blot analysis of tumor tissues from subcutaneous xenograft models showing that SAMD9 positively regulates tumor stemness in vivo. g) A lung metastatic model showing that SAMD9 positively regulates ESCC cells lung metastasis (*n* = 5/group). 1.0 × 10^6^ cells in 0.1 mL of PBS were injected into mice via the tail vein and lungs were collected after one month of cell injection. h) Animal experiments show that SAMD9 promotes ESCC relapse. When the mean tumor volume reached ∼45 mm^3^, the nude mice were treated with 5 mg/kg CDDP every 2 days for 4 times. The tumor volume was measured on the indicated days (*n* = 5 per group). Significance between the control and treatment groups was determined using an unpaired two‐tailed Student *t*‐test, and a *p*‐value less than 0.05 was considered statistically significant. Error bar, SD. ^**^, *p* < 0.01; ^***^, *p* < 0.001.

To investigate whether SAMD9 is directly involved in ESCC progression, we first measured SAMD9 expression in several cultured ESCC cell lines (Figure S1a, Supporting Information) in order to choose in vitro cell models for genetic manipulation. We chose TE1 and KYSE50 cells for SAMD9 knock‐down experiments since the parental cells showed high SAMD9 expression (Figure S1b, Supporting Information). Meanwhile, we selected ECA109 and KYSE270 cells for SAMD9 overexpression studies (Figure S1c, Supporting Information). Our results showed that overexpression of SAMD9 dramatically increased soft agar colony formation (**Figure** **2**a), sphere formation (Figure 2b), migration, and invasion abilities of ECA109 and KYSE270 cells (Figure 2c). In contrast, silencing SAMD9 by shRNA inhibited soft agar colony formation (Figure 2a), sphere formation (Figure 2b), invasion, and migration in TE1 and KYSE50 cells (Figure 2c). Consistently, the subcutaneous xenograft model results also showed that SAMD9 overexpression (Figure S1d, Supporting Information) significantly enhanced the ability of tumor formation (Figure 2d) and tumor growth (Figure 2e). In contrast, silencing SAMD9 (Figure S1d, Supporting Information) dramatically suppressed tumor formation and growth in nude mice compared to that of the vector control (Figure 2d,e). In addition, we found that cancer stemness markers, such as SOX2, Nanog, CD44, CD133, and ALDH1 were significantly upregulated in xenograft tumors overexpressing SAMD9 (Figure 2f). In contrast, shRNA knocking down SAMD9 downregulated these cancer stemness markers (Figure 2f). Furthermore, we confirmed a metastasis‐promoting effect of SAMD9 in vivo using the lung metastatic model that was generated by tail vein injection of ESCC cells with SAMD9 overexpressed. As shown in Figure 2g and Figure S2, Supporting Information, mice injected with SAMD9‐overexpressing cells had more pulmonary metastasis. Meanwhile, mice injected with SAMD9‐silenced cells had a fewer number of pulmonary metastases compared to their controls. Finally, to determine whether the level of SAMD9 is directly associated with ESCC recurrence, when the mean tumor volume reached ∼45 mm^3^, mice inoculated with ECA109‐vector and ECA109‐SAMD9 or TE1‐scramble and TE1‐shRNA of SAMD9 cells began receiving CDDP (cis‐diaminedichloroplatinum) treatment. As shown in Figure 2h, tumors xenograft with vector control cells started to regrowth after 6 days of transient reduction in mass volume, whereas tumors that expressed a higher level of SAMD9 regrowth after 3 days of transient reduction in mass volume. Notably, tumors formed by SAMD9 silenced cells showed failed to regrowth within 12 days after a transient reduction in mass volume (Figure 2h) and the body weight shown in Figure S5, Supporting Information. Together, these data indicate that SAMD9 positively regulates the tumorigenicity, stemness, and metastasis ability of ESCC cells.

### 2.3 SAMD9 Stimulates Stemness, Epithelial–Mesenchymal Transition (EMT), Angiogenesis, and MYH9 Expression through Activating Wnt/*β*‐Catenin Pathway in ESCC

**Figure 3:**
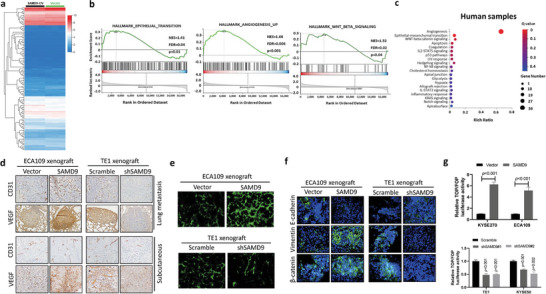
SAMD9 stimulates angiogenesis, EMT, and the Wnt/*β*‐catenin pathway. a) Differentially expressed genes between SAMD9‐overexpressed KYSE270 cells (SAMD9‐OV) and their control cells (vector). KYSE270 cells were transfected with a SAMD9‐expressing construct or empty vector for 72 h, followed by mRNA sequencing. b) The gene set enrichment analysis (GSEA) shows that SAMD9 is positively associated with EMT, angiogenesis, and the Wnt/*β*‐catenin pathway in ESCC cells. The GSEA was performed using mRNA sequencing data from SAMD9‐OV and their vector control cells. c) Encyclopedia of Genes and Genomes analysis of primary tumors mRNA sequencing dataset showing that angiogenesis, EMT, and Wnt/*β*‐catenin pathways are involved in metastatic recurrence of ESCC. The mRNA sequencing data from the primary tumor samples of ESCC patients with or without postoperative metastatic recurrence (Figure 1a). d) Immunohistochemistry analysis shows that expression of CD31 and VEGF is positively regulated by SAMD9 in tumors of animal models. Tumors from subcutaneous xenograft models (Figure 2e) and lung metastatic models (Figure 2g). e) Visualized tumor vasculature images were obtained from xenograft tumors perfused with FITC‐lectin by confocal fluorescence microscopy (*n* = 5/group). f) Immunofluorescence analysis of tumor tissues from subcutaneous xenograft models (Figure 2e) shows that SAMD9 positively regulates Vimentin and *β*‐catenin expression, while negatively regulating E‐cadherin expression. Blue: DAPI; Green: indicated proteins. g) TOP/FOP luciferase analysis shows that SAMD9 positively regulates *β*‐catenin signaling in ESCC cells. Each bar represents the mean of three independent experiments. Significance between the control and treatment groups was determined using an unpaired two‐tailed Student *t‐*test, and a *p‐*value less than 0.05 was considered statistically significant. Error bar, SD.

To elucidate the oncogenic mechanism of SAMD9, RNA sequencing was performed using SAMD9‐overexpressed ESCC cells and their control cells (**Figure** **3**a). Then, using this RNA sequencing dataset performed gene set enrichment analysis (GSEA). The GSEA results show that SAMD9‐overexpression was associated heavily with signaling involved in EMT, angiogenesis, and Wnt/*β*‐catenin (Figure 3b). Interestingly, the Kyoto Encyclopedia of Genes and Genomes (KEGG) analysis using RNA sequencing dataset of primary ESCC samples (Figure 1a) showed that Wnt/*β*‐catenin pathway, EMT, and angiogenesis are essential factors for postoperative metastatic recurrence of ESCC (Figure 3c), suggesting that SAMD9 might promote ESCC metastatic recurrence via stimulating Wnt/*β*‐catenin pathway, angiogenesis, and EMT. To prove this hypothesis, the correlation between SAMD9 and angiogenesis, EMT, or Wnt/*β*‐catenin pathway was examined in the xenograft model by measuring their marker proteins expression by the IHC. The results showed that SAMD9‐overexpression dramatically increased mean vessel density (MVD) and VEGF expression, while, silencing SAMD9 decreased MVD and VEGF (Figure 3d). Such an angiogenesis‐promoting effect of SAMD9 was further demonstrated by confocal fluorescence microscopy in the xenograft model. Our data showed that SAMD9 overexpression increased the number of blood vessels in tumors, whereas SAMD9 knock‐down decreased the number of blood vessels in tumors (Figure 3e). Additionally, the positive regulation of vimentin and *β*‐catenin expression, and the negative regulation of E‐cadherin expression by SAMD9 were demonstrated in xenograft tumor tissues by IF analysis (Figure 3f). In addition, the TOP/FOP luciferase reporter analysis confirmed that SAMD9 activates the Wnt/*β*‐catenin pathway (Figure 3g). Together, our findings suggest that SAMD9 stimulates stemness, angiogenesis, EMT, and the Wnt/*β*‐catenin pathway in ESCC.

**Figure 4:**
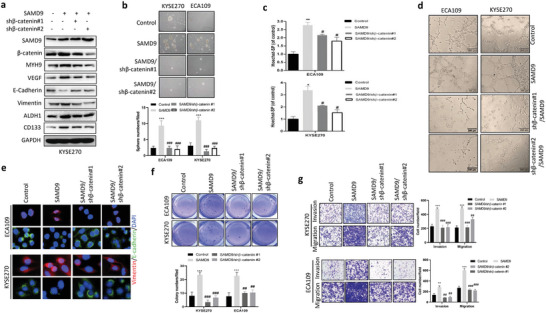
Silencing *β*‐catenin inhibits SAMD9‐induced oncogenic effects in ESCC cells. a) Western blot analysis showing that silencing *β*‐catenin inhibits SAMD9‐stimulated cancer stemness, angiogenesis, EMT, and upregulation of MYH9 in KYSE270 cells. After 72 h of transfection with the indicated constructs, cells were harvested and subjected to western blot analysis. b) Silencing *β*‐catenin inhibited SAMD9‐stimulated sphere formation of ECA109 and KYSE270 cells. After 24 h of transfection with the indicated constructs, cells were subjected to sphere formation assay. c) Flow cytometry analysis shows that silencing of *β*‐catenin inhibited SAMD9‐upregulated side population (SP) in ESCC cells. After 72 h of transfection with the indicated constructs, cells were subjected to flow cytometry analysis. d) In vitro vasculogenic mimicry tube formation assay showed that overexpression of SAMD9 promotes angiogenesis, whereas silencing of *β*‐catenin inhibits the pro‐angiogenic effect of SAMD9 in ESCC cells. After 24 h of transfection with the indicated constructs, cells were subjected to a vasculogenic mimicry tube formation assay. e) Immunofluorescence (IF) analysis showing that silencing of *β*‐catenin inhibited SAMD9‐stimulated EMT in ESCC cells. After 72 h of transfection with the indicated constructs, cells were subjected to IF analysis. f) Silencing *β*‐catenin inhibited SAMD9‐stimulated soft agar colony formation, g) invasion and migration. Sphere formation, colony formation, and transwell assays were performed after 24 h of transfection. Each bar represents the mean of three independent experiments. Significance between the control and treatment groups was determined using an unpaired two‐tailed Student *t‐*test. Error bar, SD. ^#^, compared to SAMD9‐overexpression group;*, compared to control group. ^#^, *p* < 0.05; ^##^, *p* < 0.01; ^###^, *p* < 0.001; *, *p* < 0.05; ^**^, *p* < 0.01; ^***^, *p* < 0.001.

The Wnt/*β*‐catenin pathway is a promoting factor of tumor stemness, angiogenesis, and EMT.^[^ ^12^ ^]^ Thus, we investigated whether SAMD9 promotes tumor stemness, angiogenesis, and EMT dependent on the Wnt/*β*‐catenin pathway in ESCC. The expression analysis of biomarker proteins by Western blot showed that silencing of *β*‐catenin suppressed cancer stemness, EMT, and angiogenesis promoted by SAMD9 overexpression in ESCC cells (**Figure** **4**a). The same is true for the sphere formation assay (Figure 4b), side population cells assay (Figure 4c), vasculogenic mimicry tube formation assay (Figure 4d), and IF analysis (Figure 4e). Notably, silencing *β*‐catenin abrogated soft agar colony formation (Figure 4f) and cellular invasiveness (Figure 4g) stimulated by SAMD9‐overexpression. These findings indicate that SAMD9 stimulates ESCC stemness, EMT, and angiogenesis through the Wnt/*β*‐catenin pathway. Additionally, we demonstrated that SAMD9‐induced MYH9 upregulation was abrogated by *β*‐catenin knock‐down (Figure 4a), indicating that SAMD9 stimulates MYH9 expression through the Wnt/*β*‐catenin pathway in ESCC.

### 2.4 SAMD9 Activates the Wnt/*β*‐Catenin Pathway Through Interaction with MYH9

**Figure 5:**
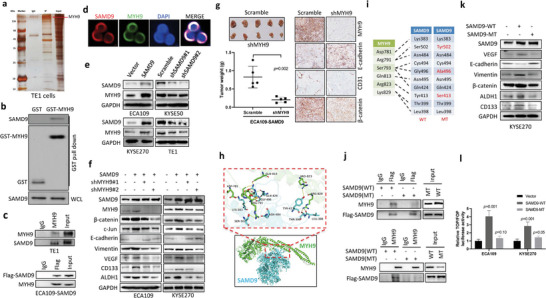
SAMD9 stimulates EMT, angiogenesis, cancer stemness, and the Wnt/*β*‐catenin pathway by binding to MYH9 in ESCC. a) Silver staining of co‐immunoprecipitation (Co‐IP) shows that SAMD9 interacts with several proteins in TE1 cells, including MYH9. Co‐IP was performed using antibodies against SAMD9. b) The physical interaction between SAMD9 and MYH9 was demonstrated by the GST pull‐down assay. c) The binding of SAMD9 and MYH9 was detected by Co‐IP in TE1 cells and Flag‐SAMD9 overexpressed ECA‐109 cells. d) Cytosolic colocalization of SAMD9 and MYH9 was detected in TE1 cells by immunofluorescence analysis. Red, SAMD9; green, MYH9, blue, DAPI. e) The positive regulation of MYH9 by SAMD9 in ESCC cells is demonstrated by WB. f) WB shows that inhibition of MYH9 blocks SAMD9‐stimulated EMT, angiogenesis, cancer stemness, and *β*‐catenin upregulation in ESCC cells. g) Silencing MYH9 inhibited tumor growth, angiogenesis, EMT, and *β*‐catenin expression in the subcutaneous xenograft model generated using SAMD9 overexpressing ECA109 cells (*n* = 5/group). h) Predicted interaction residues between SAMD9 and MYH9 using HDOCK server. i) The mutation of amino acid residues of SAMD9 (red). j) The Co‐IP analysis shows that SAMD9 mutation reduces the interaction between SAMD9 and MYH9 in ECA109 cells. k) WB analysis shows that mutation of SAMD9 did not affect EMT, angiogenesis, cancer stemness, and *β*‐catenin expression in SAMD9 overexpressing ESCC cells. After 72 h of transfection with the indicated constructs, cells were subjected to Co‐IP and WB analysis. l) TOP/FOP reporter analysis shows that mutation of SAMD9 did not activate the Wnt/*β*‐catenin pathway in ESCC cells. After 36 h of transfection with the indicated constructs, cells were subjected to luciferase assay. All in vitro data was from three independent experiments. Significance between the control and treatment groups was determined using an unpaired two‐tailed Student *t‐*test, and a *p‐*value less than 0.05 was considered statistically significant. Error bar, SD.

To elucidate the mechanism by which SAMD9 stimulates the Wnt/*β*‐catenin pathway, we identified proteins interacting with SAMD9 by co‐immunoprecipitation and mass spectrometry (**Figure** **5**a). We found that SAMD9 interacted with GSK3*β* negative regulator MYH9 (Figure 5a). The interaction of SAMD9 and MYH9 was further confirmed by GST‐pull down (Figure 5b) and Co‐IP (Figure 5c), and the colocalization of SAMD9 and MYH9 proteins in the ESCC cells was confirmed by IF (Figure 5d). In addition, we found a positive regulation of SAMD9 on MYH9 expression in ESCC cells (Figure 5e). Importantly, we found that SAMD9‐induced upregulation of *β*‐catenin was inhibited by MYH9 silencing (Figure 5f), indicating that SAMD9 activates the Wnt/*β*‐catenin pathway through MYH9. Silencing MYH9 also inhibited SAMD9‐induced cancer stemness, angiogenesis, and EMT in ESCC cells (Figure 5f). Consistently, silencing of MYH9 significantly inhibited tumor growth, angiogenesis, EMT, and *β*‐catenin expression in animal models that were generated using SAMD9 overexpressing ESCC cells (Figure 5g). These findings suggest that MYH9 plays a key role in SAMD9 activation of the Wnt/*β*‐catenin pathway.

To ensure that SAMD9 activates the Wnt/*β*‐catenin pathway by interacting with MYH9, we predicted the interaction residues between SAMD9 and MYH9 using Rosetta software (https://rosettacommons.org) and identified 10 candidate interaction residues (Figure 5h,i). Then, we constructed the mutant SAMD9 by changing the amino acid residue that was included in the interaction residues (Figure 5j). These constructs expressing flag‐tagged, wild‐type, or mutant SAMD9 were transfected into ECA109 cells and checked the interaction between MYH9 and flag‐tagged SAMD9 by Co‐IP. As shown in Figure 5j, the mutations in SAMD9 reduced the interaction of MYH9 and SAMD9 compared to the wild‐type. Importantly, the Western blot analysis showed that the mutant SAMD9 was incapable to stimulate *β*‐catenin expression, stemness, EMT, and angiogenesis (Figure 5k). Consistently, the TOP/FOP luciferase analysis showed that the mutant SAMD9 no longer activated the Wnt/*β*‐catenin pathway (Figure 5l). In addition, the promoting effect of mutant SAMD9 on soft agar colony formation (Figure S3a, Supporting Information), sphere formation (Figure S3b, Supporting Information), invasion, and migration (Figure S3c, Supporting Information) was significantly weaker than that of wild‐type SAMD9.

### 2.5 SAMD9 Activated the Wnt/*β*‐Catenin Pathway by Stimulating MYH9‐Mediated GSK3*β* Ubiquitination

**Figure 6:**
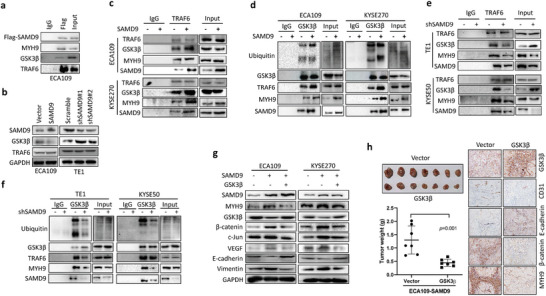
SAMD9 stimulates Wnt/*β*‐catenin pathway by stimulating MYH9‐mediated GSK3*β* degradation in ESCC cells. a) Co‐IP experiments show that SAMD9 form a complex with MYH9, GSK3*β*, and TRAF6 in ECA109 cells. b) Western blot analysis shows that SAMD9 negatively regulates GSK3*β* expression in ESCC cells. c) Co‐IP experiment show that SAMD9 increases the interaction of MYH9, TRAF6, and GSK3*β* in ESCC cells. d) Ubiquitination analysis shows that SAMD9 promotes GSK3*β* ubiquitination in ESCC cells. e) The Co‐IP experiment shows that silencing of SAMD9 reduced the interaction of MYH9, TRAF6, and GSK3*β* in ESCC cells. f) Ubiquitination analysis shows that silencing SAMD9 inhibits GSK3*β* ubiquitination in ESCC cells. g) Western blot analysis shows that overexpression of GSK3*β* inhibits SAMD9‐stimulated angiogenesis, EMT, and upregulation of c‐Jun, and MYH9 in ESCC cells. All in vitro experiments were performed after 72 h of cell transfection. h) GSK3*β*‐overexpression inhibits tumor growth, EMT, angiogenesis, and expression of MYH9 and *β*‐catenin in a subcutaneous xenograft model overexpressing SAMD9. 2 × 10^6^ cells in 0.1 mL PBS were injected subcutaneously into the back of mice (*n* = 7). One month after cell injection, mice were sacrificed and collected tumors. Significance was determined using an unpaired two‐tailed Student *t‐*test, and a *p‐*value less than 0.05 was considered statistically significant.

Since MYH9 activates the Wnt/*β*‐catenin pathway via promoting ubiquitin‐ligase TRAF6 mediated GSK3*β* ubiquitination by interactions with these proteins,^[^ ^9^ ^]^ we investigated whether SAMD9 promotes the Wnt/*β*‐catenin pathway by enhancing the ubiquitination of GSK3*β* regulated by the MYH9/TRAF6 axis. First, we used the Co‐IP experiment to find that SAMD9 forms complexes with MYH9, GSK3*β*, and TRAF6 in ESCC cells (**Figure** **6**a). We then demonstrated SAMD9 negatively regulates GSK3*β* expression in ESCC cells but does not affect TRAF6 expression (Figure 6b). Notably, overexpression of SAMD9 increased the interaction of GSK3*β*, TRAF6, and MYH9 (Figure 6c), and it promoted GSK3*β* ubiquitination (Figure 6d). In contrast, silencing SAMD9 increased the total GSK3*β* level (Figure 6b), decreased the interaction of GSK3*β*, TRAF6, and MYH9 (Figure 6e), and inhibited GSK3*β* ubiquitination (Figure 6f). In addition, SAMD9‐induced upregulation of MYH9, pro‐angiogenesis, and pro‐EMT were also abolished by GSK3*β* overexpression (Figure 6g). Consistently, overexpression of GSK3*β* significantly inhibited SAMD9‐stimulated soft agar colony formation (Figure S4a, Supporting Information), sphere formation (Figure S4b, Supporting Information), invasion, and migration in ESCC cells (Figure S4c, Supporting Information). Finally, we demonstrated that overexpression of GSK3*β* dramatically suppressed tumor growth, angiogenesis, EMT, and the expression of *β*‐catenin and MYH9 in animal models generated by SAMD9 overexpressing ESCC cells (Figure 6h). Overall, these data indicate that SAMD9 stimulates GSK3*β* ubiquitination by enhancing the MYH9/TRAF6/GSK3*β* interaction, thereby activating *β*‐catenin signaling and ultimately upregulating MYH9 expression and promoting ESCC progression.

### 2.6 Correlation between SAMD9, MYH9, Angiogenesis, EMT, and *β*‐Catenin Signaling in ESCC Patients

**Figure 7:**
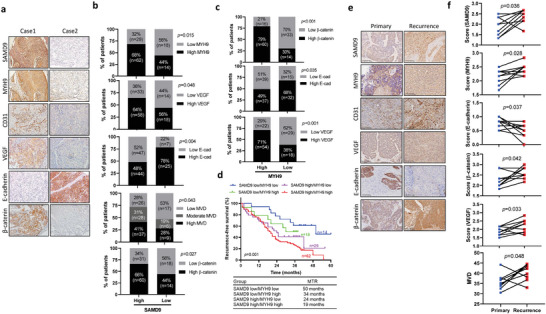
Expression correlation between SAMD9 and its target genes in ESCC clinical samples. A,b) Expression correlation between SAMD9 and its target genes was examined in 123 primary tumors of ESCC patients. Significance was determined using the chi‐square test. c) Expression correlation between MYH9 and VEGF, E‐cadherin, and *β*‐catenin was determined in 123 primary tumors of ESCC patients. Significance was determined using the chi‐square test. d) The correlation between the expression of SAMD9 and MYH9 in primary tumors and the recurrence‐free survival rate of ESCC patients was examined by Kaplan–Meier survival analysis. e,f) Expression of SAMD9 and its target genes were detected by IHC in primary and recurrent tumors of nine ESCC patients. Significance was determined using a paired two‐tailed Student *t‐*test. MVD, microvessel (CD31‐positive vessel) density.

In the clinical samples, we further confirmed the correlation among the expression of SAMD9 and MYH9 with angiogenesis, EMT, and *β*‐catenin signaling by IHC analysis (**Figure** **7**a). Consistent with the in vitro and animal experiments data, SAMD9 level was positively correlated with the level of MYH9, CD31, VEGF, and *β*‐catenin, while it was negatively correlated with E‐cadherin level in primary ESCC tissues (Figure 7b). Additionally, the IHC analysis of primary ESCC tissues showed that SAMD9 downstream protein MYH9 expression also positively correlated with the expression of CD31, VEGF, and *β*‐catenin, while negatively correlated with E‐cadherin expression (Figure 7c). In addition, compared to other groups, ESCC patients with high SAMD9 and MYH9 expression in primary ESCC tissues had the worst RFS rate and a shorter median time to recurrence (Figure 7d). Finally, we compared the expression levels of SAMD9 and its downstream genes between recurrent and their corresponding primary ESCC tissues (Table S3, Supporting Information). Our data show that SAMD9 and genes positively regulated by SAMD9, including MYH9, *β*‐catenin, VEGF, and CD31, are highly expressed in recurrent ESCC tissues compared to primary ESCC tissues (Figure 7e,f). In contrast, E‐cadherin, which is negatively regulated by SAMD9, is downregulated in recurrent ESCC tissues compared to primary ESCC tissues (Figure 7e,f).

## 3 Discussion

Here, we demonstrate that the high level of SAMD9 has potential as an indicator of recurrence and poor prognosis in postoperative ESCC patients. SAMD9 activates the Wnt/*β*‐catenin pathway, which promotes tumorigenicity and metastasis of ESCC cells. Especially, silencing SAMD9 prevented ESCC progression, suggesting that SAMD9 has potential as a therapeutic target for ESCC.

At present, surgical resection combined with neoadjuvant therapy is the mainstay of treatment for ESCC. However, recurrence after resection significantly affects the survival of ESCC patients.^[^ ^13^ ^]^ Thus, there is a need to screen for biomarkers that can identify patients with a high risk of postoperative recurrence. Here, we used transcriptomic and a series of experiments to demonstrate that SAMD9 stimulates ESCC cell metastasis and tumorigenicity. We also pinpoint the significant correlation between postoperative recurrence and high levels of SAMD9 in ESCC patients by analyzing clinical samples. Notably, multivariate analyses indicate that a high level of SAMD9 is an independent poor prognostic indicator of ESCC. Our study suggests that the high level of SAMD9 has potential as a predictor of postoperative recurrence and poor prognosis in ESCC. However, large‐sample cohorts are needed to validate our findings.

Here, we elucidated the oncogenic mechanism of SAMD9 in ESCC. EMT is a process in which epithelial tumor cells lose their cell polarity and cell‐cell adhesion and gain migratory and invasive properties, thereby contributing to the separation of tumor cells from primary tumors and moving to other sites.^[^ ^14^ ^]^ EMT also can increase CSCs and their stemness in several types of tumors.^[^ ^15^ ^]^ CSCs are a subset of cancer cells with stem cell‐like behavior within cancer tissues, they are resistant to chemotherapy, highly aggressive, and able to self‐renew and regenerate all of the cell types in the tumor, thereby contributing to metastatic recurrence.^[^ ^16^ ^]^ In addition, angiogenesis is also required for metastatic tumor formation. Because the nutrients needed for tumor cell proliferation and tumor growth are supplied by blood vessels, malignant cells must again undergo angiogenesis to result in a clinically relevant secondary tumor that eventually forms macro‐metastases.^[^ ^17^ ^]^ Importantly, studies have shown that enhanced EMT, stemness, and angiogenesis are partly associated with abnormally activated Wnt/*β*‐catenin pathways in cancer.^[^ ^18^ ^]^ In this study, we identified *β*‐catenin and its‐mediated cancer stemness, EMT, and angiogenesis as downstream signaling of SAMD9 in ESCC progression. Notably, the association between high levels of SAMD9 and EMT, angiogenesis, and the Wnt/*β*‐catenin pathway was demonstrated in the clinical specimens. These findings indicate that upregulated SAMD9 stimulates metastatic recurrence of ESCC through enhanced stemness, EMT, and angiogenesis by activating the Wnt/*β*‐catenin pathway.

We also clarified the mechanism by which SAMD9 activates the Wnt/*β*‐catenin pathway. A previous report has shown that MYH9 is a scaffold protein that recruits TRAF6 to the negative regulator GSK3*β* of the Wnt/*β*‐catenin pathway, thereby promoting the degradation of GSK3*β*, leading to hyperactivation of Wnt/*β*‐catenin pathway.^[^ ^9^ ^]^ Interestingly, the interaction of MYH9, GSK3*β*, and TRAF6 is regulated by the binding partner protein of MYH9, like HBV X protein.^[^ ^9^ ^]^ Here, our study identified a new binding partner of MYH9, SAMD9, which stimulated GSK3*β* degradation via enhancing interaction of MYH9, TRAF6, and GSK3*β* in ESCC cells, thereby, potentiated the Wnt/*β*‐catenin pathway, and ultimately stimulated tumor stemness, angiogenesis, and EMT. This study provides a mechanism by which SAMD9 activates the Wnt/*β*‐catenin pathway through stimulating MYH9/TRAF6 regulated ubiquitination of GSK3*β*.

Finally, we demonstrated the mechanism for the abnormally high expression of MYH9 in ESCC. According to a report by Lin et al., abnormal GSK3*β* degradation caused hyperactivation of the Wnt/*β*‐catenin pathway stimulates MYH9 expression by increasing c‐Jun expression in hepatocellular carcinoma.^[^ ^9^ ^]^ Here, we demonstrate that SAMD9 stimulates the expression of MYH9 and c‐Jun, and activates the *β*‐catenin pathway by downregulating GSK3*β* in ESCC. In addition, inhibition of the *β*‐catenin pathway by GSK3*β*‐overexpression or *β*‐catenin silencing dramatically inhibited SAMD9‐induced MYH9 and c‐Jun expression. Importantly, clinical samples analysis also showed that SAMD9 expression is positively associated with MYH9 and *β*‐catenin expressions in ESCC patients. These data suggest that MYH9 upregulation in ESCC is caused by abnormally upregulated expression of SAMD9 through activation of *β*‐catenin/c‐Jun signaling.

## 4 Conclusion

**Figure 8:**
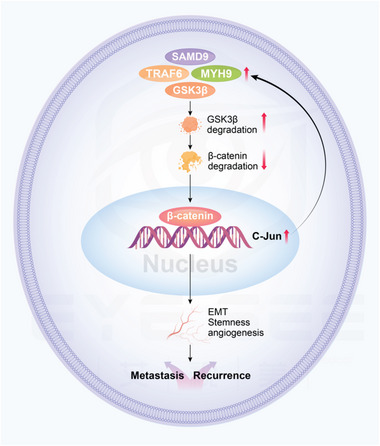
The working model of SAMD9 in ESCC.

In this study, we revealed a novel mechanism by which SAMD9 promotes the postoperative progression of ESCC through the MYH9/GSK3*β*/*β*‐catenin regulatory axis (**Figure** **8**). Further, we demonstrated that targeting SAMD9 can remarkably inhibit metastasis and tumorigenicity of ESCC cells. These data suggesting that the biological and clinical significance of SAMD9 for its potential use as a predictor for ESCC recurrence and as a target for ESCC therapy.

## 5 Experimental Section

### Cell Culture and Clinical Samples

Cell lines were obtained from the Cell Bank of the Type Culture Collection of the Chinese Academy of Sciences (TE1, ECA109, KYSE270, and KYSE50, Shanghai, China), the American Type Culture Collection (KYSE450 and KYSE70, Manassas, VA, USA) and iCell Bioscience Inc. (HECC, (Shanghai, China). Cells were maintained in RPMI‐1640 medium with 10% fetal bovine serum (HyClone, Logan, UT, USA). Primary tumor tissues were collected during the operation of 123 patients diagnosed with ESCC in Daping Hospital. Recurrent tumor tissues were collected by biopsy from nine cases. Characteristics of the patients were summarized in **Table** **1** and Tables S1 and S2, Supporting Information.

**Table 1:** Characteristics of the patients with ESCC

Variable	Total (*n* = 123)	SAMD9 expression levels		*P* (Chi‐square test)
		High (*n* = 91)	Low (*n* = 32)	
Gender				0.60
Female	23	18	5	
Male	100	73	27	
Age				0.46
60 ≤	36	25	11	
60 >	87	66	21	
Depth of invasion				0.23
T1/2	36	24	12	
T3/4	87	67	20	
Lymph node metastasis				0.04
Negative	51	33	18	
Positive	72	58	14	
Histological grade				0.81
G1	17	12	5	
G2	58	42	16	
G3	48	37	11	
Stage				0.24
I/II	62	43	19	
III/IVa	61	48	13	
Chemotherapy				0.44
Yes	61	47	14	
No	62	44	18	

### Plasmids

Plasmids expressing flag‐SAMD9 (NM_017654.4) or flag‐MYH9 (NM‐002473.6) or GST‐MYH9 were purchased from GeneCham (Shanghai, China). GSK3*β* (NM‐001146156.1) expression construct, and top‐/fop‐flash reporter constructs were purchased from GeneCopoeia (Catalog #: EX‐C0666‐M39, MD, USA) and Millipore Inc. (Catalog#:21‐170, MO, USA), respectively. The shRNA constructs against SAMD9, MYH9, and *β*‐catenin containing the puromycin resistance gene were from Shanghai GeneChem. The target sequences are summarized in Table S3, Supporting Information.

### Transwell, Soft Agar Colony, and Sphere Formation Assays

Twenty four hours after transfection with indicated constructs, cells were subjected to analysis. For soft agar colony formation assay, 1 mL of 1% noble agar (Sigma‐Aldrich, St.Louis, MO, USA) in 40 °C culture medium was added to each well of a 6‐well plate and allowed to solidify in a cell culture hood for 30 min. Cells were suspended in 0.35% agar in a growth medium and 0.5 mL of the agar‐cell mixture was plated on top of a solid layer of 1% agar (5000 cells per well). Colonies were photographed and counted 12 days later. For sphere formation assay, cells were grown in FBS‐free DMEM‐F12 (Invitrogen, Carlsbad, CA, USA), supplemented with 20 ng mL^−1^ epidermal growth factor (EGF) and bFGF and 2% B27 (Invitrogen), and plated at 1000 cells/well in ultra‐low adherent 6‐well plates (Corning, NY, USA). EGF and bFGF were added every 3 days. Two weeks later, spheres were photographed and counted. For transwell assay, 3 × 10^4^ cells in serum‐free growth medium were seeded in the upper wells of 24‐well invasion or migration chambers (Corning). The lower side of the chamber contained the growth medium with 10% FBS. After 24 h of seeding, the lower side cells of the chamber were fixed, stained, and counted.

### Side Population Cell Assay

Side population cells were detected by flow cytometry as described by Tan et al.^[^ ^19^ ^]^ Briefly, ESCC cells were transfected with indicated constructs for 72 h, and then trypsinized and resuspended in a growth medium at a concentration of 1 × 10^6^ cells mL^−1^ and incubated for 10 min at 37 °C, 5% CO_2_. Then, Hoechst 33342 (Sigma‐Aldrich) was added to the cells at a final concentration of 5 µg mL^−1^ and incubated for 90 min at dark conditions. Cells were washed and resuspended in ice‐cold HBSS+. Hoechst 33342 staining was detected using a flow cytometer (BD Biosciences, San Jose, CA, USA), exciting at 357 nm and detecting Hoechst Blue with a 424/44 broad pass filter and Hoechst Red with a 675/20 BP filter.

### Vascular Mimicry

Indicated ESCC cells were transfected with indicated constructs for 24 h, and then subjected to vascular mimicry formation assay. 300 µL matrigel was added into each well of the 24‐well plate. Then, 1 × 10^5^ cells in 300 µL complete medium (Lonza, Walkersville, MD, USA) were added to each well and incubated at 37 °C, 5% CO_2_. After 12 h of incubation and photographs were taken.

### Glutathione‐S‐Transferase (GST) Pull‐Down Assay

Plasmid for expressing MYH9‐GST and control vectors were purchased from GeneChem. The GST control or MYH9‐GST proteins were expressed in *Escherichia coli* and purified using a GST‐Tag Protein Purification Kit (Beyotime, Shanghai, China). For GST pull‐down, 500 µg of GST and MYH9‐GST fusion proteins were immobilized in 100 µL of glutathione agarose (Sigma‐Aldrich) and equilibrated before being incubated together at 4 °C for 2 h with a gentle rocking motion. 300 µg proteins containing Flag‐SAMD9 from 293T cell lysate were added to the immobilized GST and SAMD9‐GST after three washes with wash buffer. Then, incubated for 2 h at 4 °C under gentle rotation. The proteins were eluted with elution buffer (10 mM glutathione in PBS, pH 8.0) and analyzed by western blotting.

### TOP/FOP Reporter Assay

Indicated constructs and FOP‐flash or TOP‐flash were transfected into cells. 36 h post‐transfection, cells were harvested and lysed. The luciferase activity of cell lysate was measured by the Dual luciferase reporter assay system according to the manufacturer`s instruction (Promega Co., Madison, WI, USA).

### Co‐Immunoprecipitation (Co‐IP), Western blot (WB), Immunohistochemistry (IHC), and Immunofluorescence Analysis (IF)

Co‐IP, WB, and IF were performed as described previously.^[^ ^20^ ^]^ IF analysis was performed after 72 h of post‐transfection. IHC was performed as described previously^[^ ^21^ ^]^ and IHC scoring was performed by two independent pathologists without knowledge of the clinicopathological findings. A total of three fields of view under high‐powered light microscopy were randomly selected from each slide and the IHC staining intensity was scored as 0, 1, 2, and 3 (0, less than 5% cells are stained; 1, 5–25% cells are stained; 2, 26–50% cells are stained; 3, more than 50% cells are stained). The microvessel (CD31‐positive vessel) density was defined as low (< 10 vessels/0.25 mm^2^), moderate (10–40 vessels/0.25 mm^2^), or high (> 40 vessels/0.25 mm^2^). Primary antibodies against SAMD9 (ab121664), MYH9 (ab138498, ab238131), and VEGF (ab1316 for IHC) were purchased from Abcam (Boston, MA, USA). For western blot, the VEGF antibody was obtained from HUABIO (ER30607, Hangzhou, Zhejiang, China). Antibodies against SOX2(23064S), Nanog(4903S), CD133 (86781S), ALDH1(54135S), GAPDH(5174S), CD31(77699S for mouse, 3528S for human), *β*‐catenin (8480S), vimentin (5741S), ubiquitin (43124S), Flag (14793S), c‐Jun(9165S), TRAF6(8028S), GSK3*β*(12456S), myc(2276S), and E‐cadherin(14472S) were purchased from Cell Signaling Technology (Boston, MA, USA). Antibodies against CD44 (bs‐0521R) and GST (bs‐2122R) were obtained from Bioss (Beijing, China).

### Animal Experiments

Six‐week‐old BALB/c mice (Beijing HFK Bioscience Co., Ltd, Beijing, China) were used for animal experiments. For subcutaneous xenograft model, cells in 0.1 mL of phosphate‐buffered saline were transplanted subcutaneously on the backs of mice. One month later, tumors were collected and weighed. For the lung metastasis model, 1.0 × 10^6^ cells in 0.1 mL of PBS were injected into mice via the tail vein. One month later, the experiment was terminated, and lung surface tumor nodules were counted.

### Tumor Vessel Imaging

2 × 10^6^ cells in 0.1 mL of PBS were injected subcutaneously into the backs of mice (*n* = 5). After 1 month of cell injection, 100 uL of fluorescein *Lycopersicon esculentum* lectin (Fisher Scientific, Waltham, MA, USA) was intravenously injected into anesthetized tumor‐bearing mice. Ten minutes later, mice were perfused with 4% paraformaldehyde through the ascending aorta. Tumors were embedded in OCT (Sigma‐Aldrich) and sectioned. Images were acquired by confocal fluorescence microscopy.

### Statistical Analysis

Data were presented as mean ± SD. The data analysis was generated using SAS software 9.4 (SAS Institute, Inc., Cary, NC, USA), and *p‐*values less than 0.05 were considered statistically significant. Student's two‐tailed *t*‐test was used to detect significant differences between the two groups. The chi‐square test and log‐rank test of the Kaplan–Meier survival curve were used to demonstrate the expression level correlation between genes and the correlation between gene expression level and survival rate of ESCC patients, respectively. A cox proportional hazards regression model was used for multivariate survival analyses. Statistical information including sample size (*n*) and *p‐*values is described in the text or the figure legends.

### Ethics Statement

All animal experiments were performed in accordance with the ethical policies and procedures approved by the Laboratory Animal Welfare and Ethics Committee of the Army Medical University (Approval no. SYXK(YU)20170002). The use of ESCC tissues was approved by the Ethics Committee of Army Medical University (Approval no. 2019(109)). The experiments were carried out with the full, informed consent of the subjects.

## Conflict of Interest

The authors declare no conflict of interest.

## Author Contributions

H.J., and C.X.X, conceived the study and H.J., C.X.X., B.X. Q.L., H.L., F.Q.D., and R.T.W. designed experiments. Q.L., H.L., R.T.W., FQ.D, and W.G. collected patient specimens and performed clinical data analysis. X.Q.F, Y.Y.L, M.S.D., and L.T performed in vitro and animal experiments. Q.L., H.L., F.Q.D., Y.W., and R.T.W. performed statistical analysis on in vitro and animal experimental data. H.J., C.X.X., and B.X. wrote the manuscript. Q.L., H.L., F.Q.D., and R.T.W. contributed equally to this work.

## Supporting information

Supporting Information

## Data Availability

The data that support the findings of this study are available from the corresponding author upon reasonable request.
