# Supplementary material for: SAMD9 Promotes Postoperative Recurrence of Esophageal Squamous Cell Carcinoma by Stimulating MYH9‐Mediated GSK3β/β‐Catenin Signaling
Source: Adv Sci (Weinh). 2023 Feb 9;10(11):2203573. doi: 10.1002/advs.202203573 (PMC10104667; doi:10.1002/advs.202203573)

ADVANCED SCIENCE
Open Access

Supporting Information

for *Adv. Sci.*, DOI 10.1002/advs.202203573

SAMD9 Promotes Postoperative Recurrence of Esophageal Squamous Cell Carcinoma by Stimulating MYH9-Mediated GSK3β/β-Catenin Signaling

Qing Li, Hao Luo, Fu-Qiang Dai, Ren-Tao Wang, Xiao-Qing Fan, Yuan-Yuan Luo, Meng-Sheng Deng, Yulun Wang, Tan Long, Wei Guo, Bo Xu, Cheng-Xiong Xu* and Hua Jin**

Supplementary Tables

Table S1. Characteristics of ESCC patients who provided samples for RNA sequencing

Variable	Total patients (n=34)	Metastatic recurrence	No recurrence	*p* value
**Gender**				0.76
Male	15	11	4	
Female	19	13	6	
Age				0.68
≤60	12	9	3	
> 60	22	15	7	
**Histologic grade**				0.85
G1/2	23	16	7	
G3/4	11	8	3	
**Depth of invasion**				0.22
T1/2	6	3	3	
T3/4	28	21	7	
**Lymph node metastasis**				0.96
Negative	10	7	3	
Positive	24	17	7	
**Stage**				0.54
Ⅰ/Ⅱ	11	7	4	
Ⅲ/IV	23	17	6	
**Postoperative chemotherapy**				0.15
YES	14	8	6	
NO	20	16	4	

Table S2. Characteristics of ESCC patients with relapse

	When initially diagnosed as ESCC				Postoperative recurrence site
	Age	Sex	TNM stage	Combination of surgery and chemotherapy	
1	78	Female	T1N0M0	Yes	liver
2	65	Male	T3N2M0	Yes	stomach
3	68	Male	T2N0M0	Yes	liver
4	65	Male	T2N0M0	Yes	liver and stomach
5	61	Male	T2N0M0	Yes	kidney
6	53	Male	T3N1M0	yes	liver
7	54	Male	T3N1M0	Yes	liver
8	62	Male	T3N1M0	Yes	local
9	55	Male	T3N1M0	Yes	peritoneal nodule

Table S3. shRNA targeting sequences

**Gene**	**Sequences**
**SAMD9**	sh#1: 5′- TCGTACAAAGCAACCAATT-3′<br>sh#2: 5′- TCTGGTGATTATCCTAAAT-3′
**MYH9**	sh#1: 5′-CCATACAACAAATACCGCTT-3′<br>sh#2: 5′-GGTAAATTCATTCGTATCAA-3′
**β-catenin**	sh#1: 5′-AACAGTCTTACCTGGACTCTG-3′<br>sh#2: 5′-AAAGGCAATCCTGAGGAAGAG-3′
**Scramble**	5′-CAACAAGATGAAGAGCACCAA-3

Supplementary figures

Figure S1. The expression level of SAMD9 was measured by Western blot and immunohistochemistry. a) The expression level of SAMD9 was measured in several ESCC cell lines by Western blot. b) Western blot analysis showing that SAMD9 was dramatically downregulated in ESCC cells by transfection of shRNA of SAMD9. After 72h of transfection, cells were subjected to analysis. c) Western blot analysis showing that SAMD9 was dramatically upregulated in ESCC cells by transfection of SAMD9-expressing construct. After 72h of transfection, cells were subjected to analysis. d) Immunohistochemistry analysis of SAMD9 expression in tumor tissues that from indicated subcutaneous xenograft models (Figure 2d).

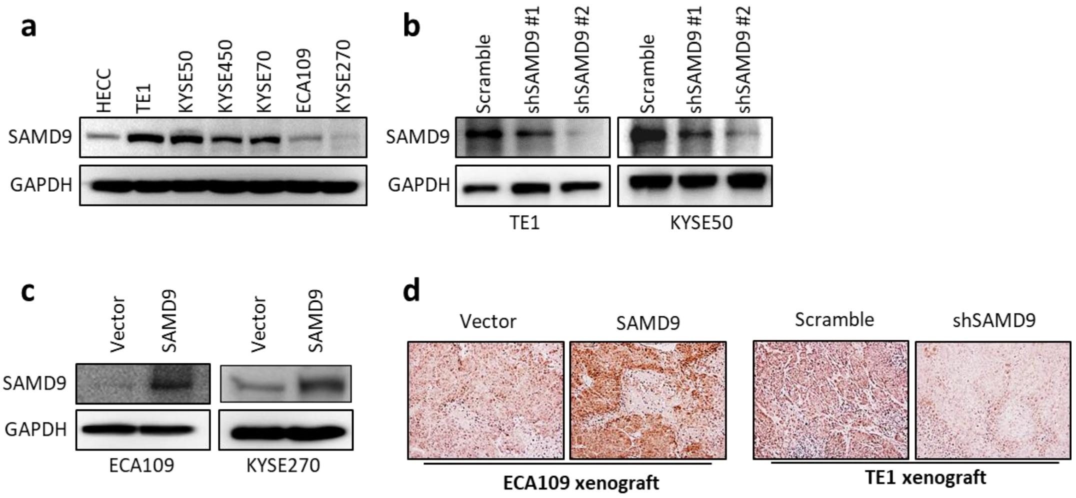

Figure S2. H&E staining showing that SAMD9 positively regulates lung metastasis of ESCC cells in lung metastatic models. a) H&E staining showing that overexpression of SAMD9 increased lung metastasis of ECA109 cells in lung metastatic models. b) H&E staining showing that silencing of SAMD9 inhibited lung metastasis of TE1 cells. Lungs were collected from indicated lung metastatic models.

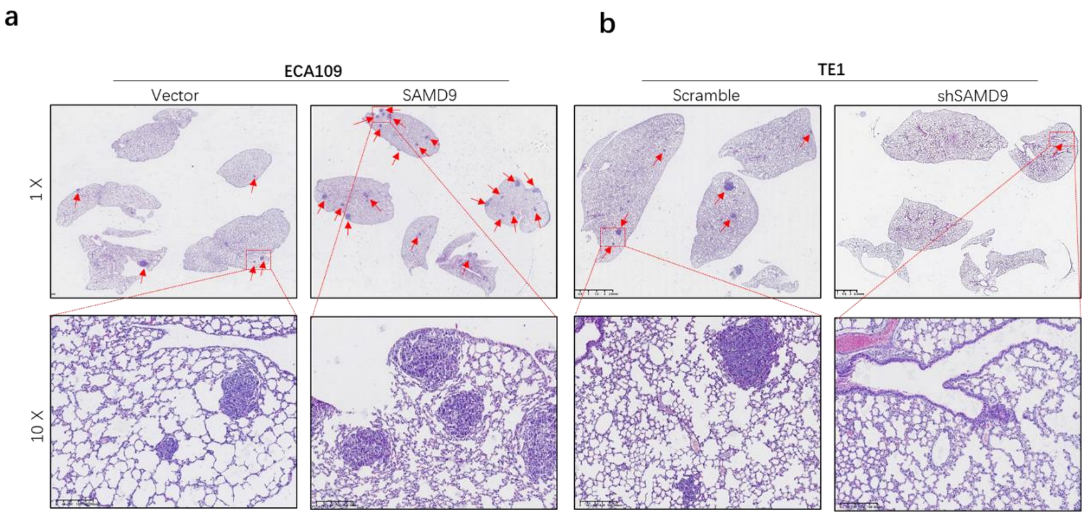

Figure S3. Overexpression of mutant SAMD9 cannot promote ESCC progression. a) Overexpression of mutant SAMD9 cannot promote colony formation in ESCC cells. b) Overexpression of mutant SAMD9 cannot promote sphere formation in ESCC cells. c) Overexpression of mutant SAMD9 cannot promote invasion and migration as significantly as overexpression of wild type SAMD9 in ESCC cells. After 24 h of transfection with indicated constructs, cells were subjected to analysis. Each bar represents the mean of three independent experiments. Significance between control and treatment group was determined using an unpaired two-tailed Student *t*-test. Error bar, standard deviation (SD). $^{\#}$, compared to wild type SAMD9-overexpression group; *, compared to vector group. $^{\#}$, $p<0.05$; $^{\#\#}$, $p<0.01$; $^{\#\#\#}$, $p<0.001$; *, $p<0.05$; **, $p<0.01$; ***, $p<0.001$.

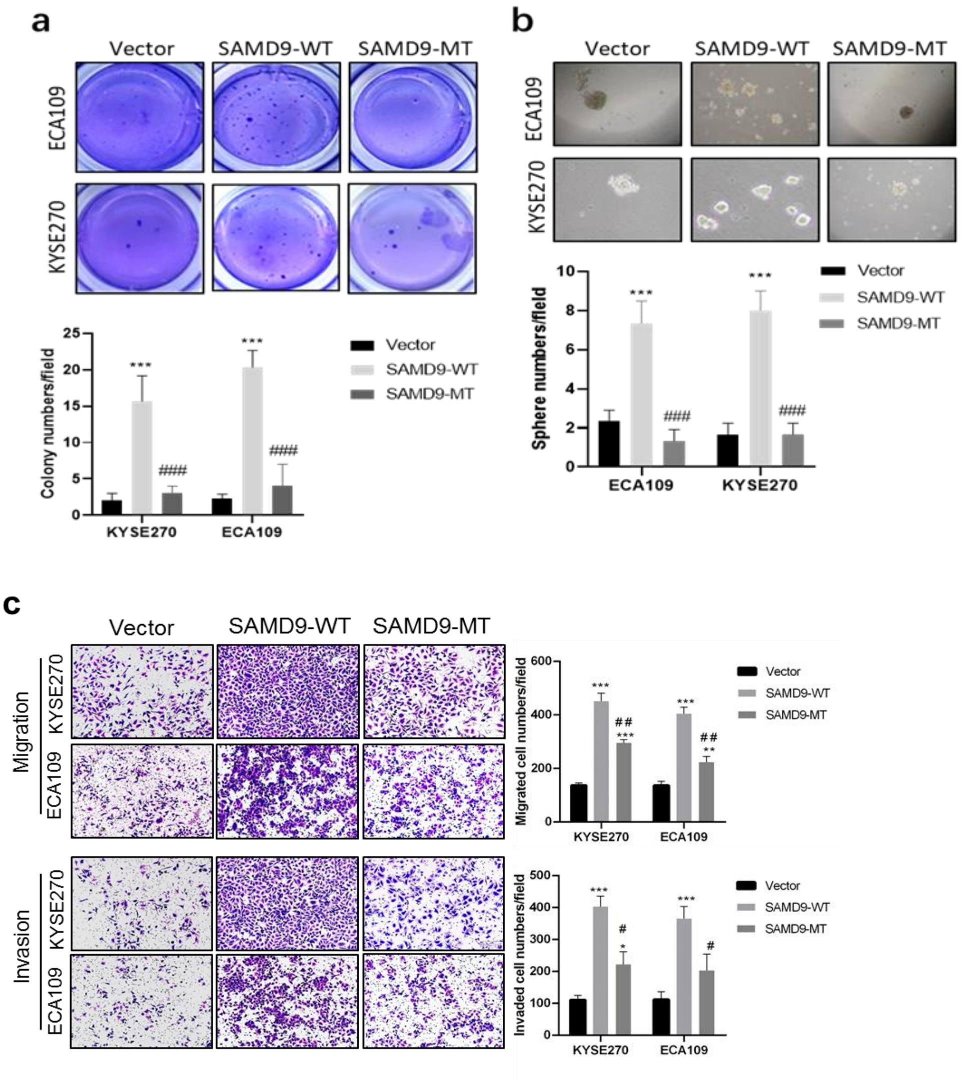

Figure S4. Overexpression of GSK3β inhibited SAMD9-overexpression stimulated colony formation, sphere formation and metastasis of ESCC cells. a) Overexpression of GSK3β inhibited SAMD9-overexpression stimulated colony formation of ESCC cells. b) Overexpression of GSK3β inhibited SAMD9-overexpression stimulated sphere formation of ESCC cells. c) Transwell analysis showing that overexpression of GSK3β inhibited SAMD9-overexpression stimulated invasion and migration of ESCC cells. All experiments were performed after 24h of transfection. Each bar represents the mean of three independent experiments. Significance between control and treatment group was determined using an unpaired two-tailed Student *t*-test. Error bar, standard deviation (SD). $^{\#}$, compared to SAMD9-overexpression group; *, compared to vector group. $^{\#}$, p<0.05; $^{\#\#}$, p<0.01; $^{\#\#\#}$, p<0.001; *, p<0.05; **, p<0.01; ***, p<0.001.

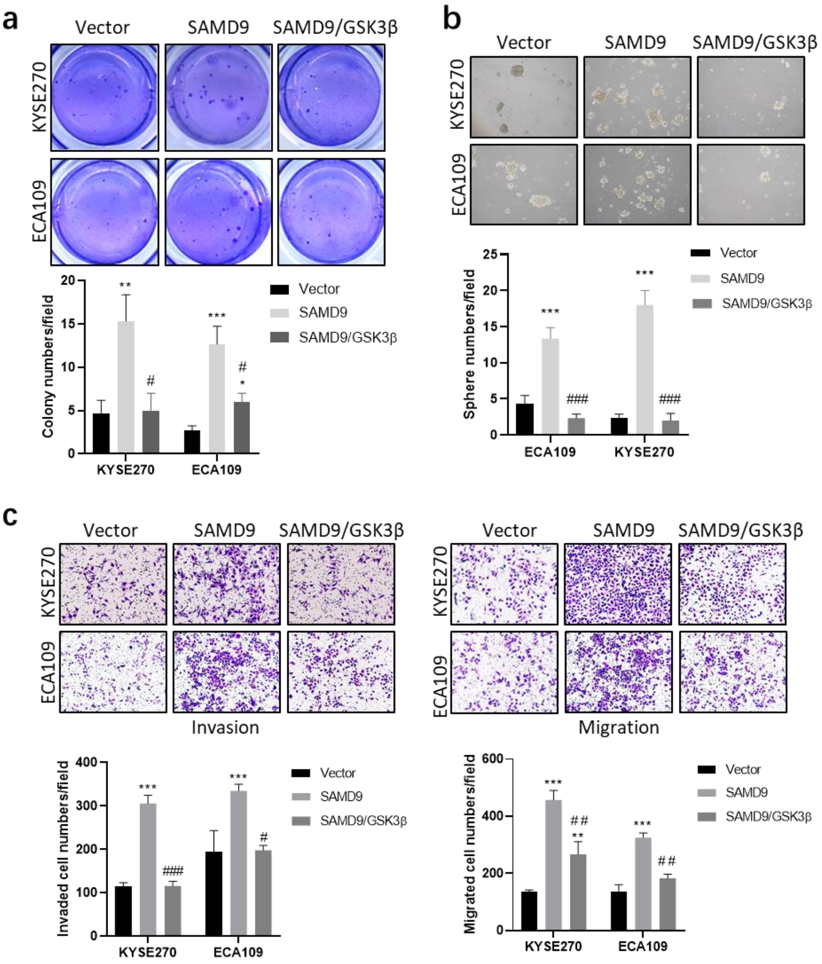

Figure S5. Body weight changes of xenograft models. Subcutaneous xenograft models were generated using indicated ESCC cells. When the mean tumor volume reached ~45mm^3, the nude mice were treated with 5 mg/kg CDDP every 2 days for 4 times. The Body weight was measured once a week (n=5 per group).

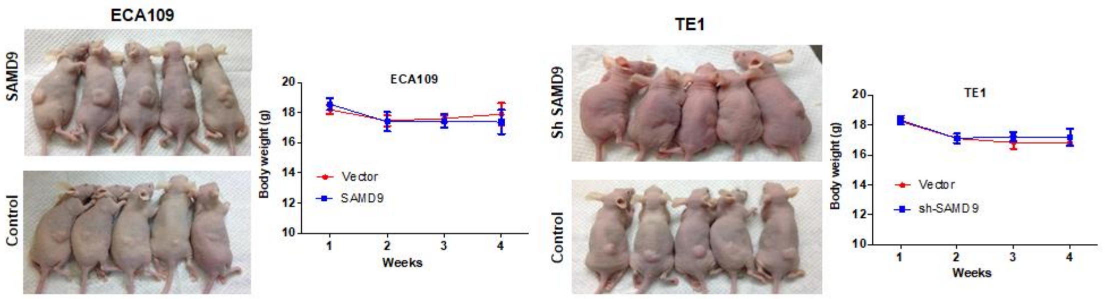

Figure S6. All Western blots that triplicated in this study.

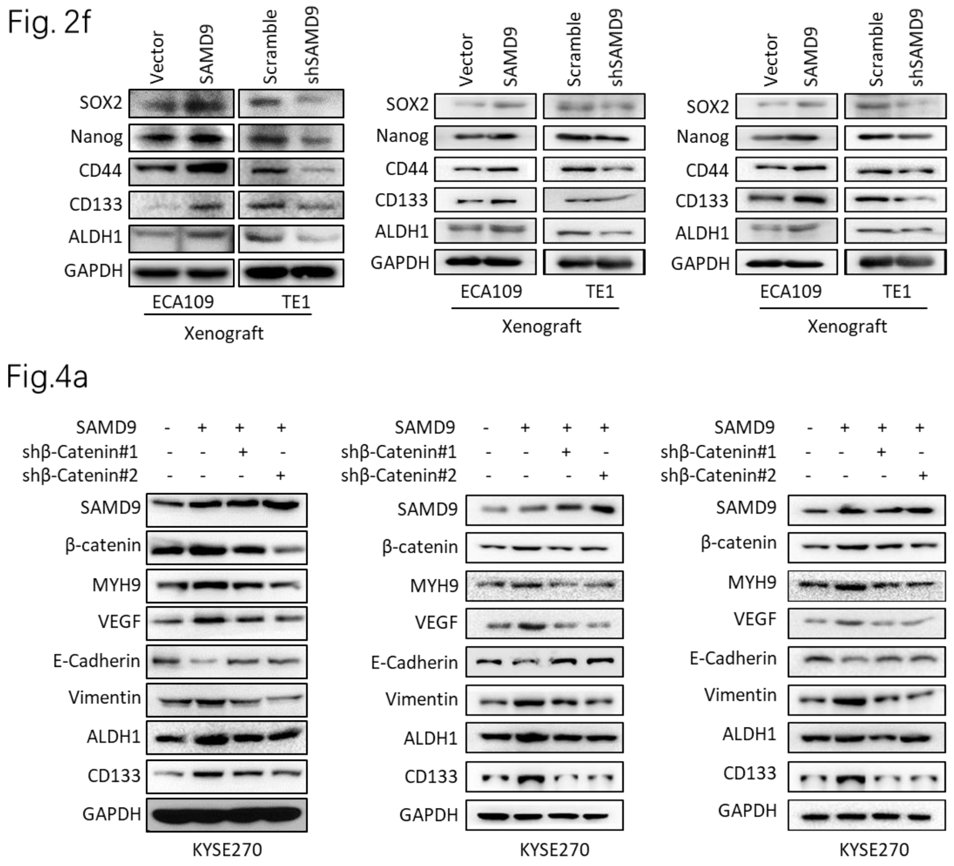

Fig. 5c

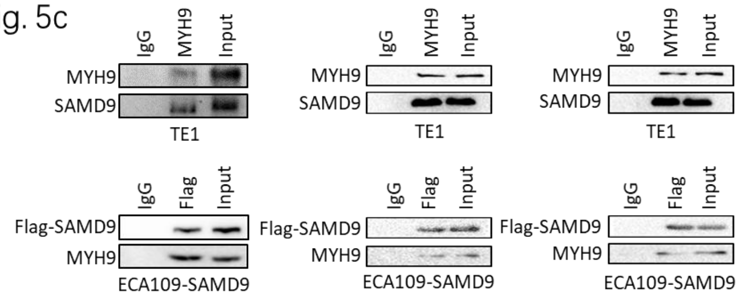

Fig.5e

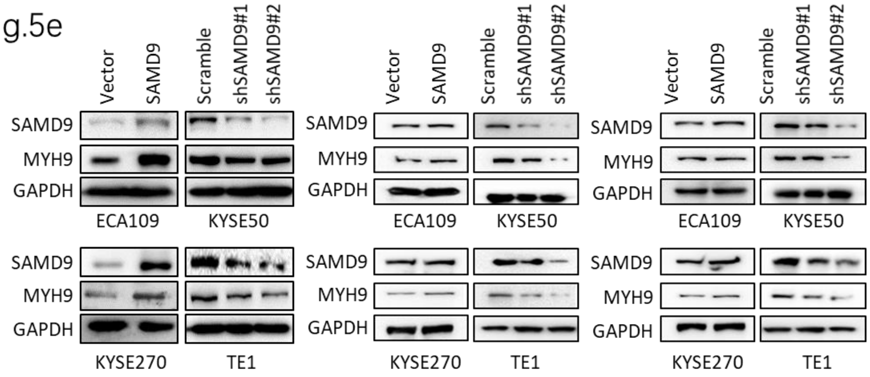

Fig.5f

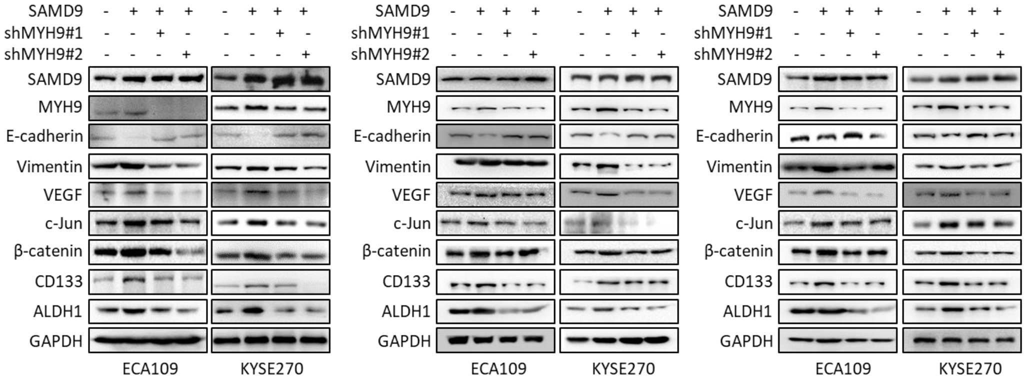

Fig.5k

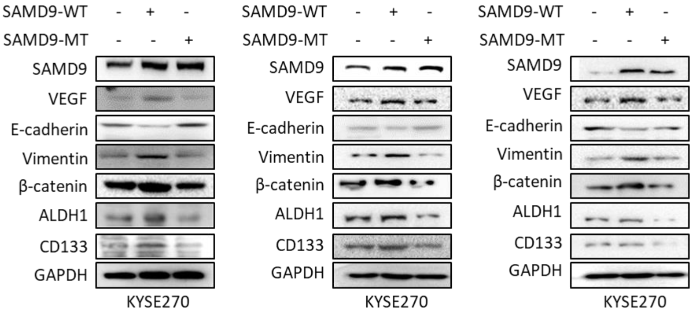

Fig.5j

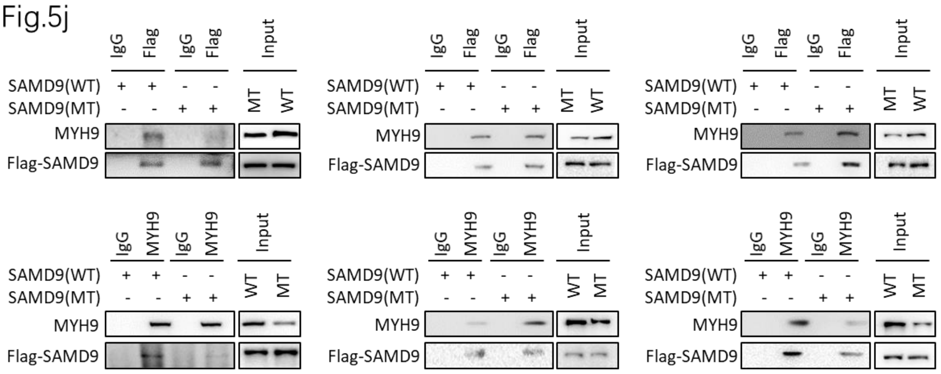

Fig.6a

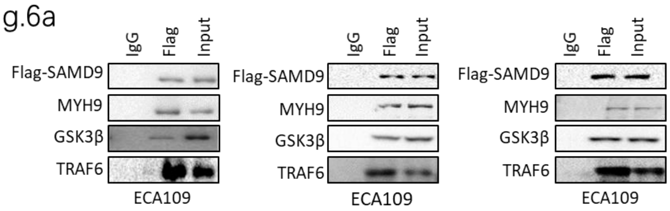

Fig.6b

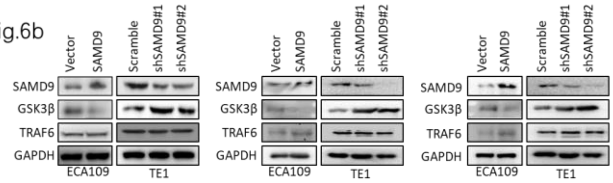

Fig. 6c

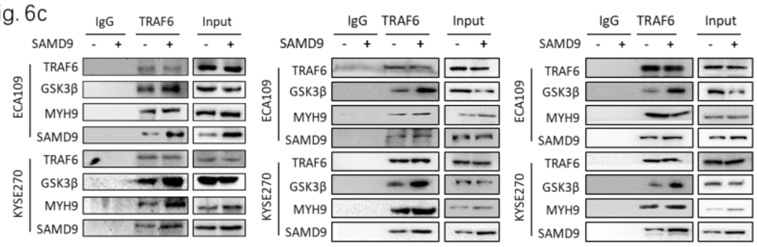

Fig. 6d

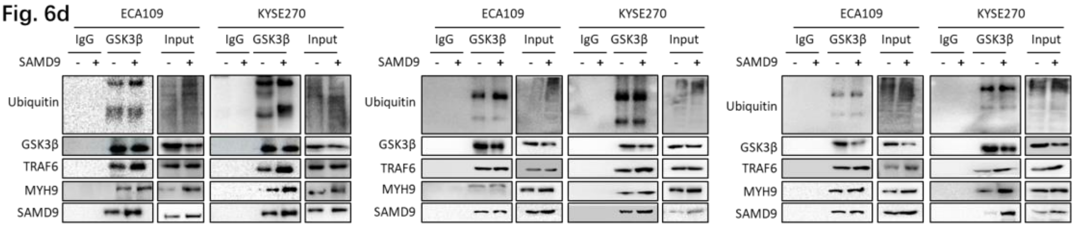

Fig. 6e

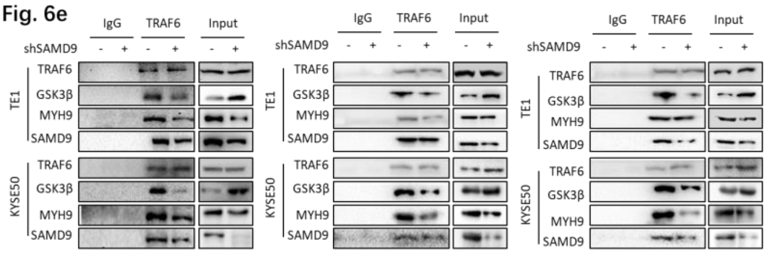

Fig. 6f

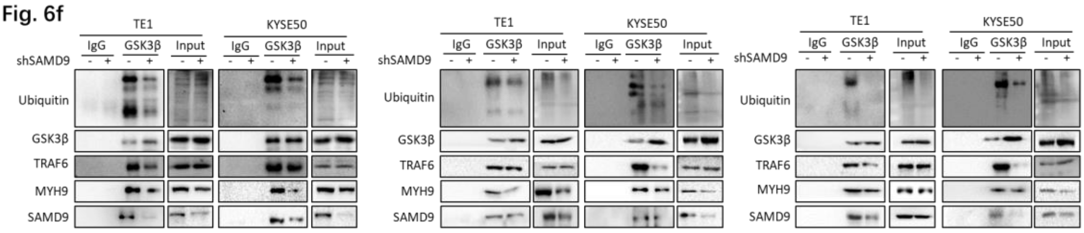

Fig. 6g

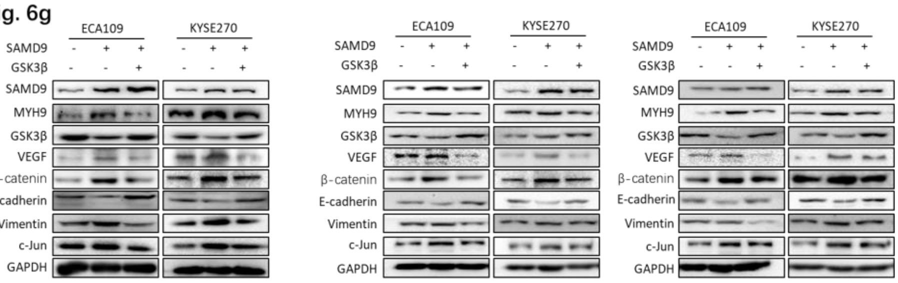

Fig. S1a

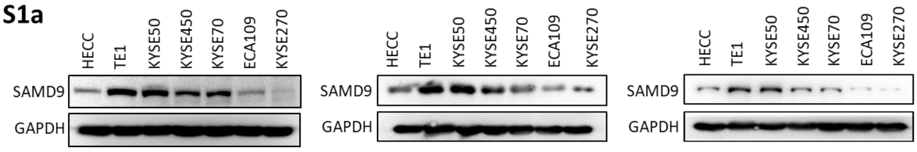

Fig. S1b

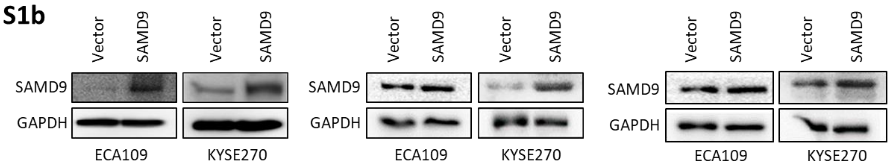

Fig. S1c

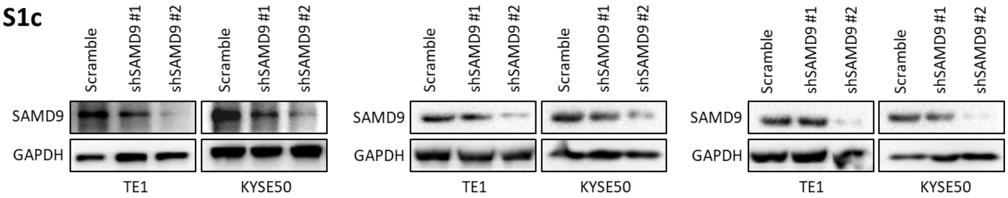

Supplement: Supplementary file 1 — Supporting Information [file ADVS-10-2203573-s001.pdf]
